# Tuberculose orbitaire: à propos d'un cas

**DOI:** 10.11604/pamj.2014.17.64.3872

**Published:** 2014-01-27

**Authors:** Zineb Khrifi, Meriem Abdellaoui, Abdellah Alaoui, Idriss Andaloussi Benatiya, Hicham Tahri

**Affiliations:** 1 Service d'Ophtalmologie, CHU Hassan II de Fès, Fès, Maroc

**Keywords:** Tuberculose, orbite, exophtalmie, tuberculosis, eye-socket, exophthalmos

## Abstract

La tuberculose sévit à l’état endémique au Maroc, l'atteinte orbitaire est rare et peut se faire par voie hématogène ou par contigüité à partir d'un foyer de voisinage. Nous rapportons le cas d'une patiente de 42 ans qui présente une exophtalmie droite dont l'enquête étiologique révèle une tuberculose orbitaire.

## Introduction

La localisation orbitaire de la tuberculose est rare, elle peut se voir dans le cadre d'une primo-infection tuberculeuse ou de lésions secondaires d'une tuberculose générale. Nous en rapportons un cas à travers lequel nous analysons les différents aspects cliniques, paracliniques et thérapeutiques de cette affection.

## Patient et observation

Il s'agit d'une patiente âgée de 42 ans, diabétique de type II sous insuline, qui consulte aux urgences ophtalmologiques pour une exophtalmie droite associée à une baisse de l'acuité visuelle du même oeil évoluant depuis un an.

**Figure 1:**
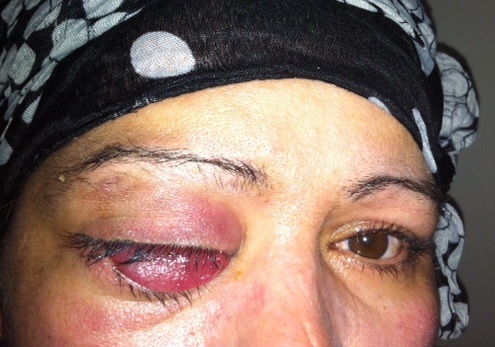
Aspect clinique de la patiente lors de la première consultation: exophtalmie inflammatoire avec chémosis conjonctival en inférieur

**Figure 2:**
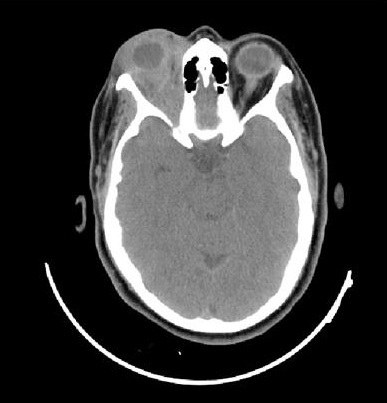
Coupe axiale d'un scanner orbitaire sans injection de produit de contraste montrant une exophtalmie droite et un processus de densité tissulaire intra-orbitaire

**Figure 3:**
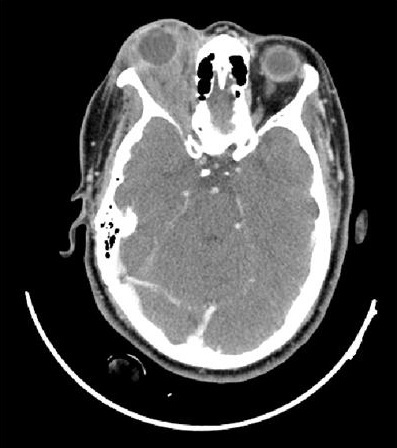
Coupe axiale d'un scanner orbitaire après injection du produit de contraste, absence de rehaussement lésionnel

**Figure 4:**
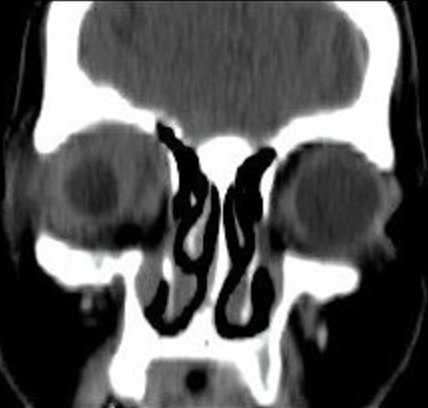
Coupe coronale du scanner orbitaire

**Figure 5:**
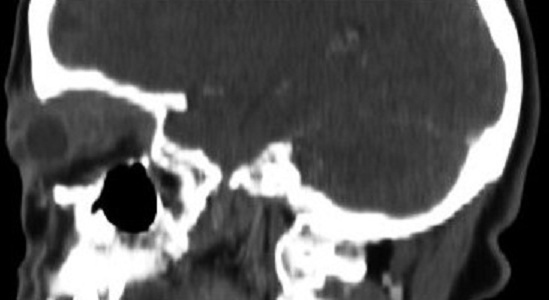
Reconstruction sagittale des coupes du scanner orbitaire

La patiente n'a pas d'antécédents de tuberculose ou de notion de contage tuberculeux. L'examen ophtalmologique trouve au niveau de l'oeil droit une acuité visuelle à 6/10ème, une exophtalmie droite non axile avec un chémosis inférieur et une masse palpable sous le rebord orbitaire supéro-externe (Figure 1), l'examen du segment antérieur et du fond d'oeil est sans anomalies. L'examen de l'oeil gauche trouve une acuité visuelle à 10/10ème avec un segment antérieur et un fond d'oeil normaux. L'examen général ne trouve pas d'autres signes extra-ophtalmologiques, notamment pas de signes respiratoires ou d'adénopathies palpables. La tomodensitométrie orbitaire objective du coté droit un processus tissulaire intra-orbitaire intra-conique et qui s’étend en supérieur, de densité tissulaire, homogène, ne se réhaussant pas après injection de produit de contraste, avec une exophtalmie homolatérale associée et sans lyse osseuse en regard ni extension intracrânienne (Figure 2, Figure 3,Figure 4, Figure 5). La biopsie de la lésion orbitaire montre un tissu fibro-adipeux remanié par un processus inflammatoire spécifique se caractérisant par la présence de plages de nécrose caséeuse accompagnées d'une réaction épithélio-giganto-cellulaire. Cet aspect histologique est concordant avec une tuberculose caséo-folliculaire.

**Figure 6:**
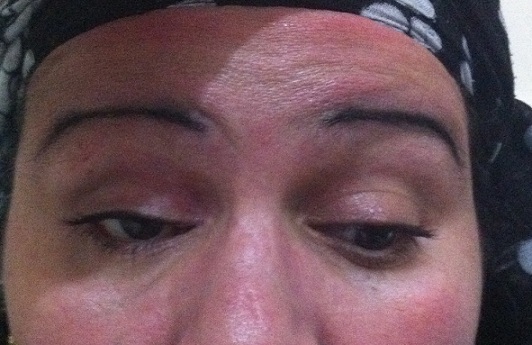
Aspect clinique de la patiente après 7 mois de traitement antibacillaire, avec disparition complète de l'exophtalmie

Un bilan comportant: NFS, ionogramme complet, VS, CRP, bilan d'hémostase, recherche de BK dans les crachats, intra-dermoréaction à la tuberculine et une radiographie thoracique est revenue normale. La patiente est mise sous traitement antibacillaire, pendant 9 mois, selon le protocole: rifampicine,isoniazide, pirazinamide et éthambutol pendant 2 mois, puis 7 mois de rifampicine et d'isoniazide. L’évolution après sept mois de traitement est marquée par la disparition complète de l'exophtalmie (Figure 6), une nette amélioration de l'acuité visuelle qui est passée à 9/10, mais la patiente a gardé une limitation du muscle droit inférieur avec une hyperaction du muscle droit supérieur droit.

## Discussion

La tuberculose est une maladie infectieuse due au bacille tuberculeux. Elle représente un grand problème de santé publique au Maroc et dans le monde entier. La tuberculose orbitaire est rare, même dans les pays où la tuberculose est endémique. En effet seuls 0,3% des patients atteints de tuberculose développent une atteinte orbitaire [1, 2]. Chez les adultes séropositifs, l'incidence de l'atteinte tuberculeuse oculaire est élevée variant entre 50 et 90%, alors que l'atteinte tuberculeuse orbitaire reste extrêmement rare [3]. L'atteinte ophtalmologique du mycobactérium tuberculosis inclut: choroidite, choriorétinite, tuberculome choroidiens, sclérite, uvéite, vascularite rétinienne, neuropathie optique et endophtalmie. L'atteinte tuberculeuse orbitaire peut intéresser aussi bien les tissus osseux que les tissus mous. Le mécanisme de l'atteinte se fait selon deux voies: directe hématologique et indirecte immunologique. [1]

La tuberculose des tissus mous peut toucher les muscles oculomoteurs et les tissus orbitaires réalisant soit une exophtalmie inflammatoire, comme c'est le cas de notre patiente, soit des troubles oculomoteurs à type de paralysie ou de limitation de la motilité par effet de masse. [1]. La tuberculose orbitaire peut aussi donner une ostéoperiostite ou un tuberculome de la glande lacrymale. Dans sa localisation extra-orbitaire elle peut donner une tuberculose du globe oculaire, ganglionnaire, pulmonaire, osseuse ou une péricardite tuberculeuse [1, 3]. L'exophtalmie secondaire à la tuberculose orbitaire peut entrainer une baisse de l'acuité visuelle, une atteinte du reflexe photomoteur, une hypertonie oculaire ou une altération du champs visuel due à une neuropathie optique compressive. Elle peut aussi conduire à une kératite d'exposition. L'atteinte orbitaire est une manifestation rare de la tuberculose, sa description provient de la publication de cas isolés ou de petites séries [1]. Seulement une cinquantaine de cas de tuberculose orbitaire ont été retrouvés dans une revue de la littérature basée sur une recherche sur PubMed prenant comme mots clés: tuberculose et orbite. Les différentes données cliniques, paracliniques et thérapeutiques de certains cas retrouvés sont résumées dans les Tableau 1, et Tableau 2.

**Tableau 1:** Revue de la littérature de certains cas de tuberculose orbitaire

Auteurs	Age/sexe	CLINIQUE	PARA-CLINIQUE	TRAITEMENT
Mahender K Narula [1]	9 cas. Ages: 1 à 15 ans;7garçons, 2 filles	-Ptosis, exophtalmie, ophtalmoplégie	-bilan biologique. -radiographie de thorax -scanner orbito-cérébral	Bonne évolution sous traitement antibacillaire
Banait S [3]	27 ans, Homme	-Exophtalmie, fièvre et altération de l’état général -L'examen ophtalmologique de l’œil droit: absence de perception lumineuse, exophtalmie non axile, Fond d’œil: atrophie optique. L'examen de l’œil gauche normal -L'examen général: adénopathies cervicales	-Sérologies:HIV positives. -TSHus: normal. -Scanner: lésion de l'apex orbitaire, étendue à la fosse cérébrale moyenne avec érosion osseuse. -Cyto-aspiration de l'adénopathie cervicale: granulome épithélio-giganto-cellulaire avec nécrose caséeuse	-Anti-bacillaires: isoniazide, éthambutol, rifampicine et pirazinamide initialeme-nt pendant 2 mois - thérapie anti-rétrovirale
Kumudini S [4]	6 ans, Garçon	-Tuméfaction palpébrale supérieure gauche et ptosis -Masse cervicale -Fièvre -Examen ophtalmologique: Normal	-TSHus: élevé à 25mUI/l -Scanner orbito-cérébral: lésion extraconique gauche -culture des cellules de la lésion orbitaire:négative -aspiration de la lésion cervicale: granulome épithélio-giganto-cellulaire	Traitement anti-bacillaire

**Tableau 2:** Revue de la littérature de certains cas de tuberculose orbitaire (suite)

Auteurs	Age/sexe	Clinique	Paraclinique	Traitement
Jamshidi M [5]	17 ans, homme	-Masse péri-orbitaire	-Biopsie+ culture: mycobactérium tuberculosis	Bonne évolution sous 6 mois de traitement anti-bacillaire
Navneet Tuli[6]	5 ans, Garçon	-Exophtalmie de l’œil gauche -fièvre -Tuméfaction cervicale	-Scanner orbito-cérébral: masse tissulaire extra-conique étendue à la fosse infra-temporale -Cyto-aspiration de la lésion cervicale et orbitotomie avec biopsie: granulome épithélio-giganto-cellulaire et nécrose caséeuse	- 2 mois d'isoniazide, rifampicine, éthambutol et pirazinamide, puis 10 mois de rifampicine et d'isoniazide -Amélioration sous traitement
Kaur A. [7]	17 ans, Homme	Exophtalmie	-scanner orbitaire: lésion orbitaire évoquant une pseudo-tumeur orbitaire	-traitement corticoïde initial:absence d'amélioration et apparition de masse orbitaire gauche et d'adénopathies cervicales -Amélioration sous antibacillaire
Owoeye JF [8]	75 ans, Femme	Tuméfaction palpébrale gauche et exophtalmie	Biopsie de la tuméfaction: tuberculose orbitaire	Régression sous traitement antibacillaire

Dans les zones endémiques, la tuberculose doit être évoquée comme étiologie de toute exophtalmie en particulier chez les patients séropositifs [3]. La sensibilisation quant aux manifestations rares de tuberculose est désormais d'une grande importance, car l′incidence de cette pathologie est en augmentation dans le monde entier en raison de l′infection à VIH et de l′immigration de personnes provenant de zones endémiques à l′Ouest [4].

Le diagnostic repose sur un faisceau d'arguments anamnestiques, cliniques, radiologiques, biologiques et histologiques. La certitude diagnostique est fournie par les prélèvements, les ponctions ou les biopsies réalisées pour étude bactériologique et histologique qui retrouve l'aspect de granulome épithélio-giganto-cellulaire avec nécrose caséeuse [4]. Le diagnostic différentiel se pose devant une exophtalmie secondaire à une pathologie endocrinienne ou non-endocrine. Chez l'adulte, l'orbitopathie thyroïdienne est la cause la plus fréquente des exophtalmies unilatérales ainsi que des exophtalmies bilatérales. D′autres causes incluent les infections non tuberculeuses, les orbitopathies inflammatoires comme les pseudo-tumeurs, les tumeurs comme les hémangiomes caverneux, les lymphangiomes et les lymphomes, et la granulomatose de Wegener.

Le traitement repose sur une poly chimiothérapie antibacillaire bien conduite alors que la chirurgie n'a pas de place, sauf pour réaliser une biopsie. Il n'existe pas de consensus sur le protocole exact de traitement antibacillaire, mais dans la majorité des cas le schéma thérapeutique pour une tuberculose isolée est 2RHZE/ 4RH soit deux mois d'association: rifampicine, isoniazide, pyrazinamide, ethambutol, et quatre mois de rifampicine et d'isoniazide [5]. Le pronostic est généralement bon en absence d'extension intracrânienne ou de terrain d'immunodépression, et en cas de prise en charge précoce et bien conduite qui permet une guérison rapide et complète [6, 7, 8]. En cas de retard de prise en charge, des séquelles peuvent s'installer comme c'est le cas de notre patiente.

## Conclusion

La tuberculose orbitaire est une atteinte rare, qui peut toucher les différentes structures que ce soit par voie hématogène ou par contigüité à partir d'un foyer de voisinage. Elle présente un grand polymorphisme clinique d'où la nécessité d’évoquer ce diagnostic devant toute inflammation inexpliquée de l'orbite.
